# Human Tissue Kallikreins-Related Peptidases Are Targets for the Treatment of Skin Desquamation Diseases

**DOI:** 10.3389/fmed.2021.777619

**Published:** 2022-03-04

**Authors:** Marcelo B. Zani, Aquiles M. Sant'Ana, Rafael C. Tognato, Jair R. Chagas, Luciano Puzer

**Affiliations:** ^1^Centro de Ciências Naturais e Humanas, Universidade Federal do ABC, Sao Bernardo do Campo, Brazil; ^2^Departamento de Biofísica, Universidade Federal de São Paulo, São Paulo, Brazil

**Keywords:** serine protease, kallikrein, inhibitor, skin desquamation, skin disease

## Abstract

Human tissue Kallikrein-related peptidases (hKLKs) are serine proteases distributed in several tissues that are involved in several biological processes. In skin, many are responsible for skin desquamation in the Stratum Corneum (SC) of the epidermis, specially hKLK5, hKLK7, hKLK6, hKLK8, and hKLK14. In SC, hKLKs cleave proteins of corneodesmosomes, an important structure responsible to maintain corneocytes attached. As part of skin desquamation, hKLKs are also involved in skin diseases with abnormal desquamation and inflammation, such as Atopic Dermatitis (AD), psoriasis, and the rare disease Netherton Syndrome (NS). Many studies point to hKLK overexpression or overactive in skin diseases, and they are also part of the natural skin inflammation process, through the PAR2 cleavage pathway. Therefore, the control of hKLK activity may offer successful treatments for skin diseases, improving the quality of life in patients. Diseases like AD, Psoriasis, and NS have an impact on social life, causing pain, itchy and mental disorders. In this review, we address the molecular mechanisms of skin desquamation, emphasizing the roles of human tissue Kallikrein-related peptidases, and the promising therapies targeting the inhibition of hKLKs.

## Introduction

The skin is the largest organ in the human body, composed of multiple layers of cells, and holds the first protection of our body against the outside world, in addition to regulation of body temperature, water loss, and production of vitamin D (1–3). Human skin covers our entire body, and it is divided into two main layers: the dermis, which is a deeper layer where resides the neurovascular supply of the skin; and the epidermis, the external layer composed mainly of keratinocytes, and the first barrier against pathogens and antigens (1–4).

Between these two layers, there is the basement membrane, a highly specialized matrix structure that separates the dermis from the epidermis through a dynamic interface. Located deeper than the dermis, there is a subcutaneous tissue containing the superficial fascia, a separating connective tissue composed primarily of collagen, and the subcutaneous fat (1).

**Figure 1:**
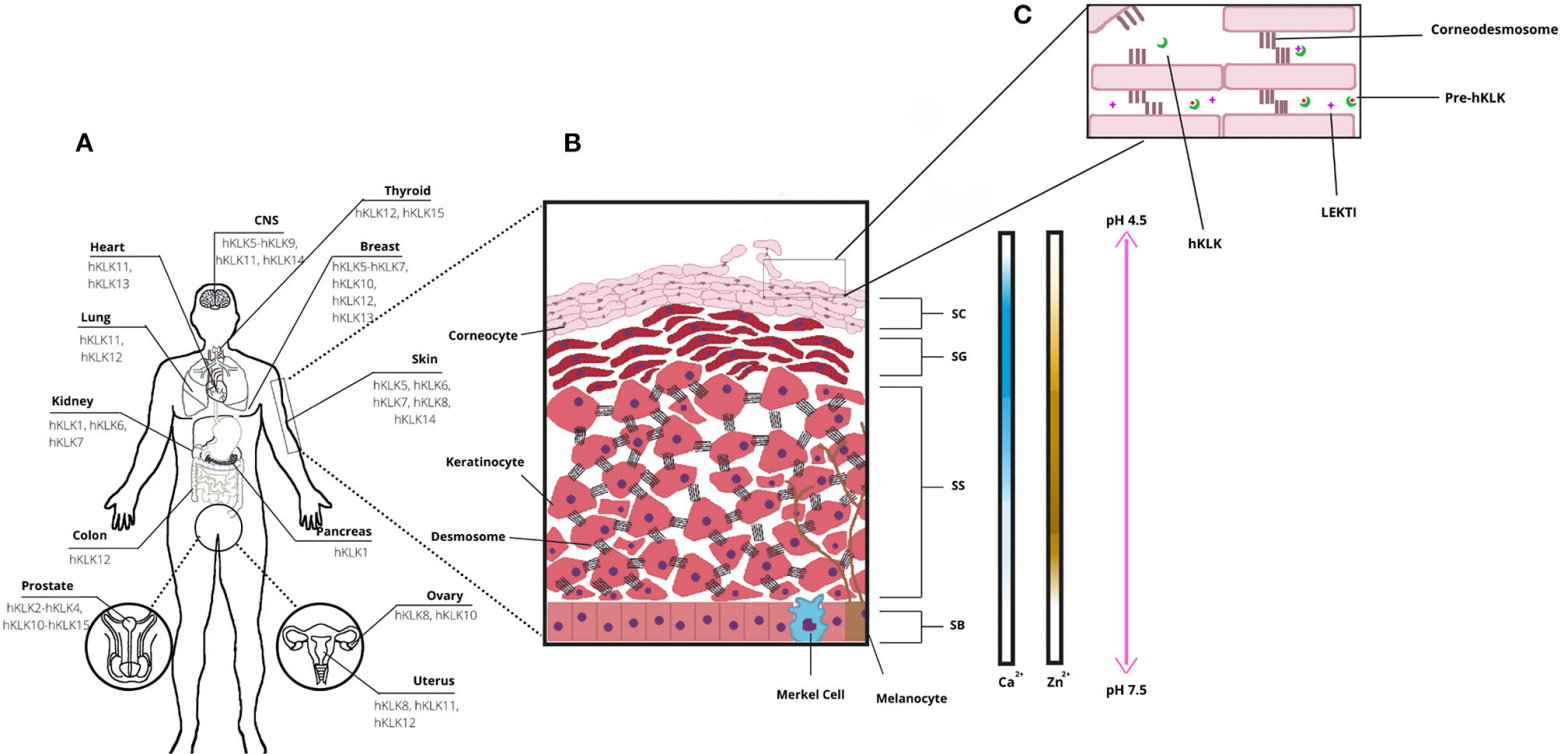
Human tissue Kallikrein-related peptidases (hKLKs) distribution and their functional role in the skin. **(A)** Distribution of the main KLKs in human tissues. **(B)** Structure of skin epidermis, the main layers, and Ca^2+^, Zn^2+^, and pH distribution, from Stratum Basale to Stratum Corneum. **(C)** Stratum Corneum (SC) and skin desquamation, a physiological process with the involvement of hKLKs, targeting the Corneodesmossome, structure responsible to maintain Corneocytes adhesion. hKLKs are expressed as a pre-enzyme not functional in deeper layers of the epidermis. Upon reaching the SC, hKLKs are activated and regulated by the natural inhibitor LEKTI. Skin desquamation is a finely regulated process in healthy subjects.

Several physiological processes occur on human skin, and they are regulated by enzymes, proteins, ions, and other signalizing molecules (3). An important physiological process is skin desquamation, which occurs at the stratum corneum on the epidermis. In desquamation, the cornified keratinocytes (corneocytes) are gradually sloughed, and this helps to maintain epidermal homeostasis and thickness, in a controlled process (5). Enzymes from the kallikrein family are directly involved in skin desquamation (Figure 1A), as the human Kallikrein-related peptidases 5 (hKLK5), 6 (hKLK), 7 (hKLK7), 8 (hKLK8), and 14 (hKLK14) (3). The deregulation on some KLKs activity caused by the mutation in the SPINK5 gene, which leads to the malformation of the endogenous inhibitor of KLKs LEKTI, is directly related to the phenotype of Netherton Syndrome, which affects the health and social life of patients. In addition, kallikreins are also identified as having a role in other skin conditions such as psoriasis and atopic dermatitis.

Deregulation of desquamation can lead to several skin diseases, such as Atopic Dermatitis, Psoriasis, and Netherton Syndrome, which are diseases that affect the health and social life of patients.

## The Structure of the Epidermis

The epidermis is composed of four distinct layers: the stratum basale (basal layer, SB), the deepest layer; the stratum spinosum (spinous layer, SS), which has a spiny appearance due to desmosome connections between keratinocytes; the stratum granulosum (granular layer, SG), named after keratohyalin granules; and the stratum corneum (SC), the outermost epidermal layer, where keratinization is completed, and desquamation occurs (Figure 1B). Keratinocytes are the most common cell type in the epidermis, spread all over the layers, and are responsible for skin keratinization (4). But they are not the only ones. In the basal layer, there are melanocytes and Merkel cells. Melanocytes are responsible for melanin production, a pigment that protects the keratinocyte nucleus from UV radiation (4–6). Melanocytes secrete melanosomes, vesicles that contain melanin, through dendritic processes and keratinocytes take them up. The pigment is distributed over the nuclei and protects the DNA from UV damage. Melanin is also responsible for skin color (6). Merkel cells are neuroendocrine cells, which associate with nerve endings to form tactile discs—structures that facilitate fine sensation most in overly sensitive locations (4). In the spinus and granular layers, there are Langerhans cells, which are antigen processing and presenting cells. They are the first immunologic defense of the skin and take the antigens to a tissue-resident macrophage, another cell type found in skin, or to naïve T-cells in lymph nodes (7).

From the deepest to the more superficial layer of the epidermis, the keratinocytes undergo the keratinization process. Initially, the stem cells in the stratum basale differentiate into keratinocytes, which start their migration to superficial layers. In the stratum spinosum, keratinocytes form desmosomes, and lamellar granules of lipids first become visible. When keratinocytes reach stratum granulosum, profilaggrin forms filaggrin inside the granules within the cells, and keratin filaments start to aggregate to filaggrin monomers, which provides a flat shape and mechanical strength to the cells (3, 8, 9). It is in the stratum granulosum that keratinocytes start to lose their organelles and become more compact. Finally, by the time keratinocytes reach the stratum corneum, the cells are named corneocytes (4). We summarize a complex and well-controlled process, and it is also important to mention that the tight balance between keratinocyte proliferation in the basal layer and desquamation at the top surface of the epidermis is essential for skin homeostasis and renewing of the stratum corneum protective barrier.

## The Role of Human Tissue Kallikrein in Skin Desquamation

The human tissue kallikreins-related peptidases form a serine protease family composed of 15 enzymes (hKLK1–hKLK15), arranged in a tandem cluster within chromosome 19q13.4. They are distributed along several tissues and are involved in many physiological processes (Figure 1A), from cellular growth regulation to tissue remodeling (10, 11). One of the most known kallikreins is the hKLK3, known as the Prostatic Specific Antigen, or PSA, a biomarker for prostatic cancer. High levels of PSA in the bloodstream may indicate the development or progression of prostatic tumors (12). Other kallikreins have been pointed as biomarkers for mammary, ovarium, or prostatic cancers, like hKLK2, hKLK5, hKLK6, hKLK10, and hKLK11 (13).

Kallikreins are secreted from cells with the help of an amino-terminal signal peptide (ranging from 16 to 34 amino acids), which targets the endoplasmic reticulum and a secretory pathway. After the signal peptide cleavage, the enzyme is an inactive zymogen that must be activated through the cleavage of another amino-terminal peptide (3).

**Figure 2:**
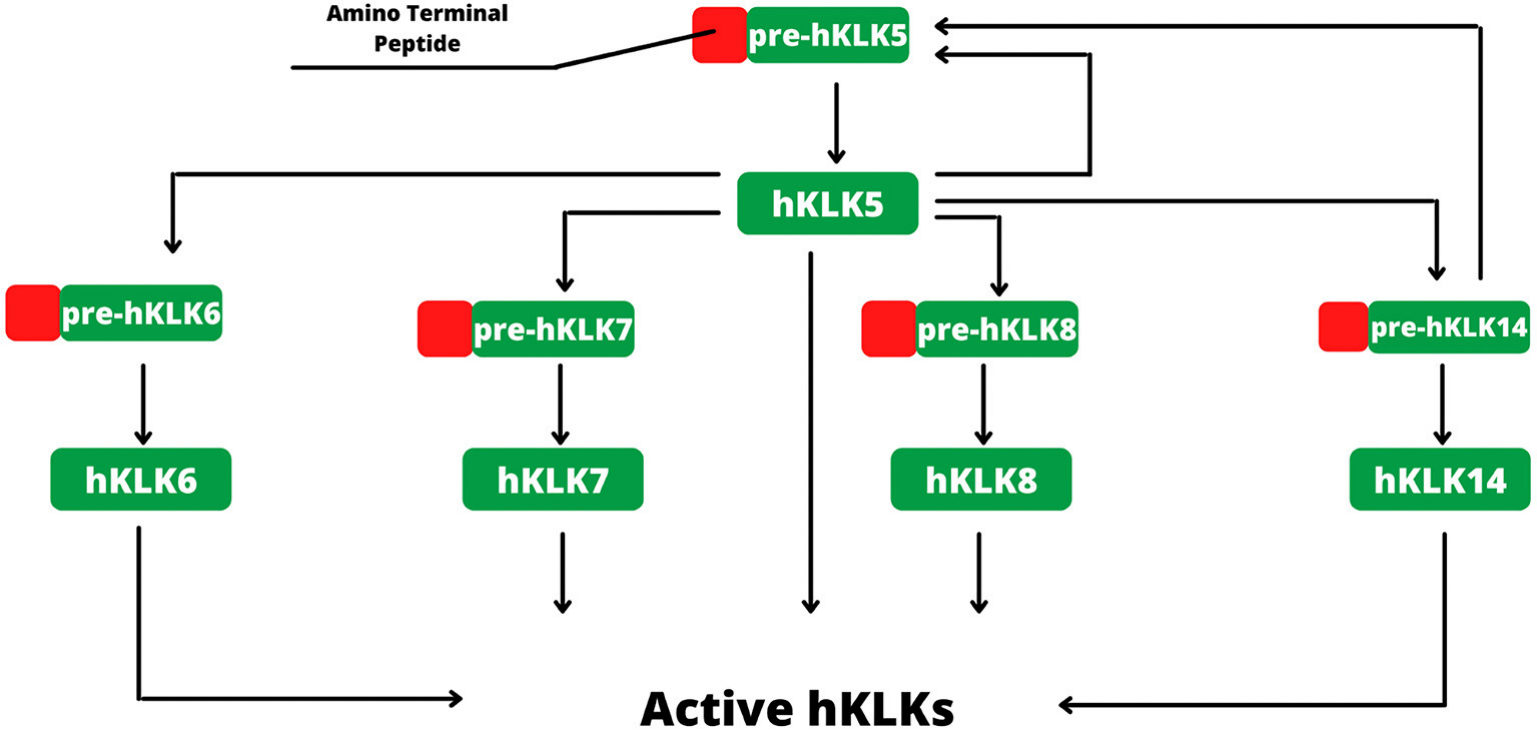
Activation of hKLKs by proteolytic cascade. The activation starts with pre-hKLK5, which is capable of self-activation. Activated hKLK5 hydrolysis pre-hKLK6, pre-hKLK7, pre-hKLK8 and pre-hKLK14. Activated hKLK14 hydrolysis pre-hKLK5, amplifying the cascade. The proteolytic cascade of hKLKs activation is regulated by endogenous inhibitors, such as LEKTI, pH, and ions.

In human skin, kallikreins are produced and secreted by keratinocytes in stratum granulosum and SC interstices (Figure 1C). The activation of zymogen occurs in a proteolytic cascade, beginning with hKLK5, which is capable of self-activation (Figure 2). Activated hKLK5 can cleave amino-terminal peptides from other kallikreins, including hKLK7 and hKLK14, both found in SC and involved in skin desquamation. Also, hKLK14 actives the hKLK5 zymogen, amplifying the proteolytic cascade (3). Such a cascade is regulated by protease inhibitors, pH, and ions (14–16). Other hKLKs are also expressed in skin, such as, hKLK4, hKLK6, hKLK8, hKLK9, hKLK10, hKLK11, and hKLK14, but the desquamation process seen to be more related to hKLK5, hKLK6, hKLK7, hKLK8, and hKLK14 (3).

Regarding the natural inhibitors, the lympho-epithelial Kazal-type-related inhibitor (LEKTI) is the main inhibitor of the kallikreins found in skin, expressed by the *SPINK5* gene (Serine-protease inhibitor Kazal-type 5) (3). LEKTI is composed of 15 serine-protease inhibitor domains (D1–D15), and each domain shows inhibitory activity. hKLK5 and hKLK14 seem to be the main targets of LEKTI, with strong inhibitions. On the other hand, hKLK7 is poorly inhibited by LEKTI and can degrade fast its domain, suggesting that another inhibitory pathway is more important to regulate hKLK7 (14). Other endogenous inhibitors are involved in the regulation of the hKLKs activity, secretory leukocyte protease inhibitor (SLPI) and elafin, both capable to reduce the shedding of corneocytes, and therefore seen to be part of the desquamation regulation process (17). SERPINs, serine protease inhibitors proteins, are also found in skin, such as SERPINB3, SERPINB4, and SERPINB13 (18). This class of proteins inhibits serine protease by a unique mechanism. An external loop (the Reactive Center Loop–RCL) of the SERPIN mimics a protease substrate, luring the target. During cleavage, the RCL changes its conformation to a β-strand inserted into a β-sheet at the center of the SERPIN structure, dragging the protease and changing its tridimensional structure. Such conformational change distorts the active site, inhibiting the protease, which is yet covalently bound to SERPIN (19, 20).

Tissue kallikrein-related peptidases of the skin can maintain their activities in a broad pH range, although the optimal activity is achieved in neutral pH (3). Such characteristic is important for their biological role, since the SG and the deep layer of SC, where the hKLKs are produced, have neutral pH, but acidification occurs in outer layers of SC, from 7.5 to 4.5 (15). As desquamation occurs in the outer layers of SC, it is only possible for a remaining activity of the hKLKs in such a pH range. LEKTI can bind to kallikreins more effectively at a neutral pH, but during skin acidification, a dissociation occurs (14). So, in the outer layers of SC, the hKLKs are more active than in deeper layers. Skin acidification is due to secretions of sebaceous and sweat glands and is seen to be related to inhibition of bacterial and fungi growth (15).

Calcium ions (Ca^2+^) are present in the epidermis, but not evenly distributed through skin layers. Rather, they form a concentration gradient from SB to SC. Concentrations of calcium are low in SB and SS layers, increase in SG, and decline again in SC (3). Morizane et al. have demonstrated that calcium induces the expression of hKLK5 and hKLK7, in normal human epidermal keratinocytes (NHEK), and also affects the activities of such enzymes. It up-regulates and down-regulates the activity of hKLK5 and hKLK7, respectively (21). But calcium is also responsible for the induction of LEKTI, SLPI, and elafin expression. Therefore, the presence of these inhibitors declines from SG to SC, and the hKLKs increase the corneodesmosome cleavage in the upper layers of SC, occurring skin desquamation (16).

Zinc is another important ion in human skin (Zn^2+^), the third most Zn-abundant tissue in the human body (22). In the epidermis, as Ca^2+^, Zn^2+^ is not evenly distributed through layers, and it has upper concentrations in SS. Zn^2+^ has many physiological roles in the human body and human skin, such as keratinocyte proliferation and suppression of inflammation (22). Also, Zn^2+^ inhibits hKLK5 activity, by binding into an allosteric domain adjacent to the active site, coordinated by imidazole rings of His57, His96, and His99 (23). Neutral pH is particularly important for hKLK5 inhibition by Zn^2+^, as such effect drops considerably when pH changes from 7.5–8.0 to 5.5. Also, the addition of EDTA to the hKLK5-Zn^2+^ complex restored hKLK5 activity, demonstrating that the inhibition by Zn^2+^ is reversible (23). As Zn^2+^ concentration is higher in SG and pH is neutral, Zn^2+^ binds to hKLK inhibiting the zymogen. After migrating to SC, pH acidifies, and Zn^2+^ concentration drops, so hKLK5 starts self-cleaving and the proteolytic cascade (22).

The role of hKLKs in skin desquamation is regarding the cleavage proteins responsible for cell adhesion. Three proteins are the targets of hKLKs, desmoglein I (DSG1), desmocollin I (DSC1), and corneodesmosin (CDSN), that together form the corneodesmosome, a modified desmosome formed in SC during the terminal differentiation of keratinocytes, and responsible for cell–cell junction (3, 24). hKLK5 can cleave all three proteins from the corneodesmosome, and hKLK7 can cleave DSC1 and CDSN (24, 25). These two peptidases are the main responsible for skin desquamation in SC, but hKLK6 and hKLK14 also showed the capability to cleave DSG1 and may also play a role in desquamation (26). Besides, a few other peptidases seen to be related to skin desquamation as well, by controlling hKLK activity, like Matriptase, a type II transmembrane serine protease that is an activator of epidermal kallikreins, meprin β, a secreted metalloendopeptidase that activates hKLK7, and the Keratinocyte-specific mesotrypsin, which activates hKLKs and also degrades LEKTI (27, 28).

Deregulation in hKLK activity results in deregulation in the skin desquamation process, which leads to a phenotype of Netherton Syndrome, and secondarily can contribute to the phenotype of other skin diseases, such as Atopic Dermatitis, Psoriasis (3).

## Skin Diseases and Their Relationship With hKLKs

Skin inflammation is deeply related to the immune response against pathogens and antigens that attack our system. Skin diseases lead to inflammation and kallikreins-related peptidases are directly involved in inflammatory pathways (3). Once the epidermal barrier is compromised, keratinocytes produce and secrete several cytokines, including interleukins 8 (IL-8) and 1 (IL-1), and tumor necrosis factor alpha (TNF-α), which causes T-lymphocytes migration (29). Inflammation is responsible for the first symptoms of skin disease: itching, redness, swollenness, warmth, and pain.

The role of kallikreins in inflammation seems to be related to Proteinase-activated receptors (PARs), a transmembrane G-coupled receptor found in different cell types, including keratinocytes (30). In keratinocytes, PARs regulate skin homeostasis, growth, differentiation, pigmentation, and cytokines release. Cleavage of PAR leads to conformational change and release of intracellular calcium, therefore signalizing downstream pathways (30, 31). In keratinocytes, cleavage of PAR2 receptor increases inflammatory cytokines release, such as IL-8, IL-13, ICAM-1 (intercellular adhesion molecule 1), TNF-α, and TSLP (thymic stromal lymphoprotein), and hKLK5, hKLK6, and hKLK14 showed *in vitro* PAR2 cleavage activity (30, 32). Also, these three hKLKs were capable to cleave PAR1 receptors *in vitro*, but only cleavage by hKLK14 showed intracellular calcium signaling response (32). After PAR cleavage and cytokines release, T-lymphocytes and other immune responsive cells migrate to the damaged skin site (3). Deregulation in kallikrein-related peptidases activity in skin diseases may induce not only acute desquamation but also inflammation through the PAR pathway.

### Atopic Dermatitis

Atopic Dermatitis (AD) is a chronic inflammatory skin disease that affects up to 20% of children and 3% of adults worldwide, and its prevalence increased by 2 to 3 times in industrialized countries in the twentieth century (33, 34). AD is more prevalent in children than in adults, and most of the patients develop the disease in the first 5 years of life (33). Although AD is not commonly persistent after the child reaches adulthood, up to 30% of patients continue to have symptoms for the rest of their lives, and therefore must stay under treatment (33). Like other skin diseases, AD has not only economic impacts, but also an impact on the quality of life (35), due to pain, itching, and mental distress.

The molecular basis of AD is not fully known yet, but genetic seems to be an important factor, more precisely the *FLG* gene, located on chromosome 1q21.3 (36). Mutations in FLG with loss of function are present in several AD patients in Europe and Asia, and they have also been related to other inflammatory diseases, such as allergy, asthma, and dermatitis (34, 37). FLG is expressed as the large phosphorylated 435 kDa protein profilaggirn (proFLG), stored in keratohyalin granules inside keratinocytes. Proteolysis of FLG results in natural moisturizing factor (NMF), low-molecular weight compounds responsible for SC hydration by absorbing atmospheric water (3). Hydration is important to maintain the healthy structure of the skin, and mutations in FLG can cause skin barrier dysfunction, allowing the entrance of allergens particles, triggering inflammatory response (2), including those related to hKLKs.

But disfunction in FLG as a trigger for hKLKs mediated inflammatory response is not the only way that hKLKs may be involved with AD development. It has been reported that AD patients have overexpression of hKLKs in SC, and higher serine protease activity in AD lesions (38). Besides, a mutation in the *SPIKN5* gene, responsible for inhibitor LEKI expression, was found to underregulate hKLKs activity in the skin and might be associated with AD symptoms (39), although it is not widely accepted (40). The 3′UTR 4 bp variant of the hKLK7 gene has also been reported as a variant with prolonged mRNA half-life (41). Such events could result in higher expression of hKLK7, and, with other factors, the development of AD.

hKLK7 has been reported as the most abundant hKLK in AD lesions, and research conducted with mice showed that KLK7 is related to AD chronic itchy even without the inflammatory process (38). Therefore, hKLKs as targets to treat AD can be highly successful, either to avoid exacerbated inflammatory response, to attenuate peptidase activity, or to replace a malfunction inhibitor.

### Psoriasis

Psoriasis is a chronic, inflammatory, skin disease, and just like AD has a high impact on quality of life due to pain, excessive skin desquamation, itching, and psychological stress (3, 42). Psoriasis has been indicated as a major global health problem, due to the number of people affected worldwide. In Europe and North America, Psoriasis prevalence is about 2%, being more common in adults than in infants (43). Most of the patients have mild Psoriasis that can be controlled with topical medication, but about 20% have severe symptoms (43). The most common form of Psoriasis is a chronic plaque-type named Psoriasis Vulgaris, with reddish, pruritic plaques covered in silvery scales (44). Psoriasis is a multifarious disease involving genetic and environmental factors, and psoriatic individuals have higher risks to develop other chronic diseases. The main characteristics of Psoriasis are changes in almost all cutaneous cells, epidermal acanthosis, hyperkeratosis, and parakeratosis (44).

The immune response in Psoriasis has been widely studied. Th1 cells seem to be the most related T cell involved in the inflammatory response in psoriatic lesions (45). Interferon-γ (INF- γ), IL-2, and IL-12 are the main cytokines released by Th1 cells and are responsible for the recruitment and activation of other immune cells, like monocytes, dendritic cells (DC), and endothelial cells (42). Keratinocytes in Psoriasis have an abnormal differentiation, which is likely due to apoptosis inhibition by INF- γ, which leads to an absence of a well-defined SG layer (46). The role of human Kallikrein-related peptidases is yet to be completely resolved, but a study comparing healthy subjects and patients with Psoriasis showed aberrant levels of hKLKs in psoriatic lesions (47). hKLK6, hKLK8, hKLK10, and hKLK13 were also in higher levels at the serum of patients with untreated Psoriasis, and a correlation between the levels of these Kallikreins and Severity Index was reported (47). hKLK6 and hKLK8 are upregulated in psoriatic lesions (47), and it was demonstrated that hKLK8 is important to the development of Munro's microabscess in the epidermis, a structure formed by an increased number of T-lymphocytes, macrophages, mast cells, and neutrophilic granulocytes (43). Munro's microabscess aggravates psoriatic lesions by IL-36 expression (43).

Experiments with mice showed an increase in KLK6, KLK7, and KLK8 mRNA levels when Psoriasis-like lesions were induced (48). However, the same experience conducted in KLK8 knockout mice did not result in KLK6 and KLK7 mRNA upregulation, suggesting a determinant role of KLK8 in proteolytic cascade in Psoriasis (48). As PAR2 is a target to hKLKs, including hKLK6 (49), and is also related to the secretion of inflammatory cytokines, the proteolytic cascade exacerbation in Psoriasis may be the trigger to the inflammatory response, or at least to positive feedback. Therefore, hKLKs are important targets to treat Psoriasis and control symptoms.

### Netherton Syndrome

Netherton syndrome (NS) is a hereditary autosomal recessive inflammatory skin disease, characterized by continuous skin desquamation, severe atopic manifestations, and hair abnormality (3, 50). NS is a rare disease, affecting 1:200,000 people worldwide, with severe symptoms, usually accompanied by dehydration and infections, due to uncontrolled desquamation (50). Mutations in the *SPINK5* gene have been reported as the main cause of NS pathophysiology, resulting in defects in LEKTI inhibitor (51). Elevated serum IgE and IgG4 levels, and increased proinflammatory cytokines in the skin have been reported in patients with NS (52, 53). Deficiency in antibodies against bacterial polysaccharides has also been reported as an NS phenotype (54), and a study comparing immune cells in NS patients and healthy subjects showed increased levels in B cells and naïve B cells, and lower levels in memory B cells in patients (55).

*SPINK5* gene is located at chromosome 5q32 in a cluster that includes *SPINK6, 7, 9, 13*, and *14* (56), and suffers alternative splicing in human keratinocytes, producing three identified transcripts (*SPINK5f-1, SPINK5sh*, and *SPINK5l*) (50, 57). Although the three transcripts have similar expression patterns, the transcript *SPINK5f-1* is about 40-folds more expressed (57). LEKTI inhibitor is expressed as a polypeptide containing 15 domains, all capable to generate the typical Kazal hairpin structure. The polypeptide is processed several times by proteolytic cleavage, generating five bioreactive fragments (50). *In vitro* experiments show that all fragments are capable to inhibit SC serine proteases, including hKLK5, hKLK7, and hKLK14 (14).

KLK5 and KLK7 seem to be the major players in skin desquamation and inflammation by the PAR-2 pathway occurring in NS (58), confirmed by experiments with mice overexpressing KLK5 (59). Both KLK5 and KLK7 downregulation activity are necessary to restore the non-NS phenotype (60). The severity of the disease is related to the degree of serine protease activity and residual LEKTI expression (61).

*SPINK5*^−/−^ mice display phenotypes related to NS and are useful tools to understand the disease and its molecular aspects. Zingkou et al. (62) studied the role of KLK6 in NS symptoms, using mice *SPINK5*^−/−^ and *KLK6*^−/−^. The results showed that KLK6 is involved with the expression of TSLP, TNF-α, and IL-23, all pro-inflammatory cytokines. Also, the double KLK6 knockout mice displayed normal keratinocyte differentiation, but proteolytic activities and excess desquamation persisted, and mice died due to severe epidermal barrier defect, similar to only *SPINK*^−/−^ mice. Finding methods to control hKLK5 and hKLK7 activities, and other skin Kallikrein-related peptidases, is necessary to controlling NS symptoms and improving a patient's quality of life.

### Other Skin Diseases

Involvement of hKLKs in other inflammatory skin diseases has been reported, such as in Acne Rosacea (AR) (3). AR is characterized by persistent centrofacial erythema, occasionally involving papulopustules and telangiectasia (spider veins), and seems to be caused by a dysregulation in the immune system, with the involvement of antimicrobial proteins called cathelicidins, more specifically the hCAP18 (63). Cathelicidins are stored in neutrophils and lamellar bodies of keratinocytes and are part of the innate immune response of the skin (63). hCAP18 is secreted in its inactive form and can be cleaved by hKLK5 into shorter peptides, such as LL-37, which signalizes an inflammatory reaction responsible for the innate immune response of the skin (64). AR patients overexpress hCAP18, which results in an increase in serine protease activity and inflammatory responses (65). But not only hCAP18 is overexpressed, but AR patients also express higher amounts of Toll-like Receptor 2 (TLR2), which causes a calcium-dependent release of hKLK5 from keratinocytes (66). The higher amounts of hKLK5 aggravate AR symptoms (65).

Dandruff, and its more severe form, seborrheic dermatitis, is a skin disease that affects the scalp, forehead, face, shoulders, and upper chest, and back. Dandruff is characterized by skin desquamation, itchy and erythema, and has been related to three etiologic factors: sebum, *Malassezia* yeast, and individual sensitivity (67). Oleic acid has been described as the main factor that triggers dandruff, and it is a product of the hydrolyzation of sebum triglycerides (67). Lipases produced by *Malassezia* hydrolyze the triglycerides present on skin sebum, producing oleic acid and other free fatty acids. *Malassezia* yeasts are found in healthy skin of all individuals, but dandruff and seborrheic dermatitis patients seem to have disruptions in the SC barrier, allowing oleic acid penetration (67). Treatment with ketoconazole-based topical antifungal improved inflammation symptoms associated with dandruff and seborrheic dermatitis but did not decrease the total fungi amount in scalp skin, indicating other mechanism pathways are more related to disease progression (68).

Expression of LEKTI is increased in dandruff, probably due to the increase of serine protease activity, and also the production of inflammatory cytokines (69, 70). Kallikrein-related peptidases have important roles in skin inflammation and desquamation, as described previously. Experiments with mice treated with oleic acid showed elevated mRNA levels of KLK7, which could be related to aggressive desquamation seen in dandruff (71). *KLK5*^−/−^ mice presented with a dandruff pattern when treated with oleic acid on the skin, but no visible signs of inflammation, indicating that KLK5 might be related to inflammation development in dandruff patients, but not to the disease development itself (71). Nevertheless, controlling inflammation in skin diseases is an important step since inflammation provokes pain, itching and impacts quality of life.

Itching is one of the most prominent symptoms that affect patients with different skin diseases such as AD, psoriasis, seborrheic dermatitis, uremic pruritus (in patients under hemodialysis) (3, 67, 72). Itchy skin is related to opioid receptors (OR) distributed in the epidermis, whereas the μ-opioid-receptor (MOR) acts as an itching trigger, and the κ-opioid-receptor (KOR) acts as itching suppressor (73). Kupczyk et al. (74) showed that psoriatic skin of patients with high itchy intensity has lower levels of KOR in the epidermis. Similar results were found in AD patients (75). Nevertheless, the use of a KOR agonist by uremic pruritus patients reduced itching compared to a control group (76), which indicates that not only KOR receptors are downregulated but also endogenous KOR agonists. As many endogenous opioids are peptides, cleavage by hKLKs is possible, resulting in increased itching, although more studies are needed.

hKLK6 and hKLK7 were found to be overexpressed in cutaneous melanoma, an aggressive skin cancer derived from melanocytes found in the skin (77). The overexpression of these kallikreins appears to be related to pre-metastatic melanoma, since hKLK6, hKLK7, hKLK8, and hKLK13 have decreased transcription and translation as the metastasis emerges (78). This suggests that hKLKs have specific roles in the epithelial-mesenchymal transition. In fact, hKLK7 overexpression induces the production of CD146 (cluster of differentiation 146) (79), also known as melanoma cell adhesion molecule (MCAM), a surface glycoprotein that functions as a receptor for laminin subunit alpha 4, a matrix protein expressed in vascular walls, which could be related to melanoma metastasis (80). *KLK6*^−/−^ mice induced with carcinogenesis developed fewer tumors compared to wild-type animals, suggesting that KLK6 has a role in skin tumor development and progression (81). In contrast to other diseases, melanomas are not related to excessive skin desquamation yet see the participation of kallikrein-related peptidases in their formation and progression, making them interesting targets for melanoma treatments.

Peeling Skin Syndrome type 1 (PSS1) is another severe genodermatosis showing an exacerbated epidermal desquamation and skin inflammation. PSS1 displays various clinical similarities with Netherton syndrome, however, its phenotype is associated with the mutations in the *CDSN* gene encoding a corneodesmosomal protein. Although there is insufficient data indicating that the proteolytic activity of kallikreins plays a role in PSS1, it was reported elevated tissue KLKs levels in the skin and serum of patients with PSS1 (82). It was also showed that KLK14, one of the endogenous ligands of PAR2, was overexpressed in a murine model of PSS1 (Cdsniep^−/−^ mice) (83).

## Therapeutic Approaches Focused on Human Tissue Kallikreins-Related Peptidases

In the past decade, there was a lot of interest in the development of proteolytic inhibitors against human tissue Kallikrein-related peptidases. There are several inhibitors synthesized from small organic compounds, derived from different classes, such as natural isocoumarins (84), isomannides (85), 3-acyltetramic (86), 1,2,4-Triazole (87), coumarin-3-carboxylate (88), Pyrido-imidazodiazepinones (89), 1,3,6-trisubstituted 1,4-diazepan-7-ones (90), imidazolinylindole (91). Besides the high specificity of these inhibitors, some of them have IC_50_ of the order of nM, many of them are not selective, presenting crosslink inhibitions with other hKLKs and other serine proteases. However, due to the deregulation of the proteolytic cascade on skin diseases, the capability of an inhibitor to act in a wider range of enzymes, especially the hKLK5, hKLK7, and hKLK14, could be an interesting feature for the restoration of the epidermal barrier, but none of these inhibitors listed were tested *in vivo* yet.

Recently, two studies described inhibition of hKLK5 in the order of μM of two lipids classes from natural extracts: ceramides (92) and triterpenoids (93). Additionally, some proteic inhibitors were also described. Chen et al. (94) have been developing inhibitors based on the scaffold of the SFTI, a small Bowman-Birk inhibitor characterized for its bifunctional inhibition of trypsin and chymotrypsin. Laureano et al. (95) used phage display to generate recombinant antibody fragments with inhibitory potential against hKLK7, presenting IC_50_ of the order of nM.

There are also biotechnology companies investing in the development of inhibitors of the hKLKs. The pharmaceutical company Novartis AG have invested in patents covering inhibitors for hKLK7 to treat diseases related to such enzyme. One of the patents covers the use of hKLK7 modulators specifically designed using the crystal structure of the enzyme and its binding pocket (96), and another one covers the use of cyclic depsipeptides to inhibit hKLK7 (97). The natural inhibitor LEKTI has also been investigated as a pharmaceutical compound to treat diseases related to hKLKs, especially Netherton syndrome. The small company Azitra Inc. developed engineered microbes to express and secrete LEKTI fragments to treat wounds of Netherton syndrome (98). SERPINs also provide a powerful tool to inhibit serine proteases, including hKLKs. Med-Discovery, a swiss franc biotechnology startup, developed specific SERPINs to inhibit hKLK5, hKLK7, and hKLK14, that could be used to treat skin disease, with the main target Netherton syndrome (99).

Another approach that has appeared in the recent works focused on skin disease treatment is to modulate gene expression locally. Di et al. (100) performed transduction through a lentivirus-based vector encoding the SPINK5 gene in epithelial grafts. On the Phase I clinical trials LEKTI was detected after 6 months of application, but the expression was significantly lower than healthy tissue. Muzumdar et al. (101) demonstrated in SPINK5 knockout mice that the genetic activation of the transcription factor nuclear factor-like 2 (Nrf2) restored the epidermal barrier function, changing the NS phenotype. Its activation induces the expression of secretory leukocyte protease inhibitor (SIpi), a potent inhibitor of the hKLK7 and the elastase 2 (Ela2). Table 1 summarizes the main approaches on skin diseases related to human tissue Kallikrein-related peptidases.

**Table 1:** Kallikrein inhibitors with potential therapeutic use.

**Type/Class**	**Compound**	**Targets**	**Reference**
Synthetic inhibitors	Isocoumarin	hKLK5 and hKLK7	(82)
	Isomannide	hKLK5 and hKLK7	(83)
	3-acyltetramic	hKLK5 and hKLK7	(84)
	imidazolinylindole	hKLK7	(89)
	1,2,4-Triazole	hKLK5, hKLK7, hKLK14, and Matriptase	(85)
	coumarin-3-carboxylate	hKLK5, hKLK7, hKLK14, and Matriptase	(86)
	pyrido-imidazodiazeoiones	hKLK7	(87)
	Modulator organic compounds	hKLK7	(94)
	cyclic depsipeptides	hKLK7	(95)
Lipids	Ceramides	hKLK5	(90)
	Triterpenoids	hKLK5	(91)
Proteic inhibitors	SFTI	hKLK5, hKLK7, and hKLK14	(92)
	Antibodies	hKLK7	(93)
	LEKTI	hKLK5, hKLK7, and hKLK14	(96)
	SERPINs	hKLK5, hKLK7, and hKLK14	(97)
Gene therapy	Lentivirus vectors	LEKTI	(98)

## Conclusion

Human tissue Kallikrein-related peptidases are involved in several biological processes, including skin desquamation and inflammation. Nevertheless, deregulation of hKLKs activity is involved in the development of skin diseases such as Atopic Dermatitis, Psoriasis, Netherton syndrome, acne rosacea, and others. Therefore, hKLKs are attractive targets for drugs and treatments, and new approaches are arising every year. Although there are still unknown factors about how hKLKs are involved in such diseases, controlling their activities in the skin is a pathway to improve symptoms and quality of life.

In cases like Netherton syndrome, an orphan disease where the natural inhibitor is compromised, investing in therapies to restore Kallikrein inhibition is essential. But such a strategy could be highly successful in other skin diseases since hKLKs are part of the inflammatory response. Drugs targeting the Kallikrein-related peptidases are promising approaches to treat and control the most common and rare skin diseases.

## Author Contributions

MZ wrote most of the text. AS'A prepared Table 1, and all research about the main inhibitors already described. RT prepared the figures. JC is a senior professor who contributed with many suggestions to improve the article. LP is the co-ordinator and main reviewer of the text. All authors contributed to the article and approved the submitted version.

## Conflict of Interest

The authors declare that the research was conducted in the absence of any commercial or financial relationships that could be construed as a potential conflict of interest.

## Publisher's Note

All claims expressed in this article are solely those of the authors and do not necessarily represent those of their affiliated organizations, or those of the publisher, the editors and the reviewers. Any product that may be evaluated in this article, or claim that may be made by its manufacturer, is not guaranteed or endorsed by the publisher.
